# Low-cost, open-source, variable speed and incline treadmill for studying impacts of neonatal locomotion

**DOI:** 10.1016/j.ohx.2020.e00097

**Published:** 2020-01-21

**Authors:** Mitchell Williams, Stuart Sater, Colin Burkhalter, Stephen Schoonen, Jacob Miller, Dev Shrestha, Michele R. Brumley, Nathan R. Schiele

**Affiliations:** a University of Idaho, Department of Mechanical Engineering, Moscow, ID, United States; b University of Idaho, Department of Biological Engineering, Moscow, ID, United States; c Idaho State University, Department of Psychology, Pocatello, ID, United States

**Keywords:** Treadmill, Open source, Low cost, 3D printing, Locomotion, Neonatal, Rat, Mouse, Arduino, Touchscreen

## Abstract

There is a need for a small-scale, laboratory treadmill to investigate impacts of neonatal locomotion on neuromuscular and musculoskeletal development in small animal models. Adult mice and rats are routinely assessed using commercially available treadmills, but these treadmills can be relatively expensive and they may lack features needed to evaluate developing animals. Therefore, to overcome these limitations, a new treadmill was designed, built and calibrated. This open-source treadmill was designed specifically for neonatal and postnatal mice and rats, and it fits within a neonatal incubator. By using predominantly off-the-shelf and 3D printed components, and a microcontroller, this treadmill was low cost and easy to reproduce. The design also included variable incline, and a transparent belt and enclosures for video and gait analysis. A touchscreen interface provided user-friendly control over belt speed and run time. Moreover, validation experiments showed high accuracy in belt speed control, allowing for tightly regulated experimental conditions. Overall, this new low-cost, open-source, variable speed and incline treadmill can be used to advance understanding of neonatal locomotion, and neuromuscular and musculoskeletal development.

## Graphical abstract

**Figure:**
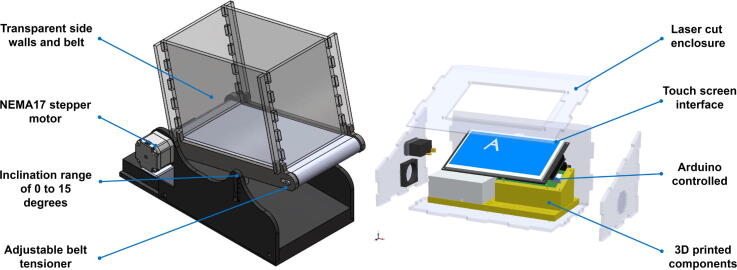

**Specifications table:** 

Hardware name	Open-Source Neonatal Treadmill
Subject area	• Medical (e.g. Pharmaceutical Science)
Hardware type	• Animal behavior and physiology • Animal development
Open Source License	GNU General Public License v3
Cost of Hardware	$388.24 USD
Source File Repository	https://osf.io/d26ku/files/

## 1 Hardware in context

Injuries to tendons are common and result in long-term dysfunction [1], [2], [3], [4], [5]. These issues have motivated studies to understand the factors that influence tendon injury in adults. One potential factor is overuse injury. To explore the role overuse plays in tendon injuries, rodent (i.e., mouse and rat) models of overuse have been developed using treadmill running [6], [7], [8], [9], [10]. The treadmills used in these studies have been modified from human treadmills [11], or have been commercially purchased systems designed for small animals. While these systems work well for understanding impacts of exercise in adult animal models of tendon injury, they are relatively expensive and not appropriate or easily adaptable for studies in developing animals.

Understanding the impacts of mechanical stimuli on musculoskeletal tissue formation during embryonic and neonatal development is of great interest [12], [13]. Recent work has explored how paralysis [14] and the onset of locomotor behavior impacts tendon formation in the developing embryo and neonate, respectively [15]. Furthermore, physical activity by the developing neonate has long-term beneficial impacts on muscle mass and levels of inflammatory cytokines at later ages [16]. To improve understanding of how mechanical stimuli from exercise influences tendon and musculoskeletal tissue formation, there is a need for treadmill systems that can be used with neonatal rodent models. Treadmill exercise is advantageous since it encourages consistent physiologically relevant locomotor behavior, and the duration, intensity, rate and timing of the exercise can be controlled [17], [18]. However, many of the current treadmill systems do not have enclosures designed for young animals or lower operational speeds. Furthermore, they do not fit inside a standard bench-top neonatal incubator. Another limitation of many treadmill systems is the lack of a transparent belt design or enclosure, which are needed for taking video and gait analysis [19].

To address the need for a small-scale treadmill, a custom treadmill system had been developed [20]. However, to our knowledge, no open-source treadmill systems appropriate for neonatal and postnatal rodent models have been described. Therefore, the goal of this design was to provide a low-cost, open-source, variable speed and variable incline treadmill for application in studies exploring neonatal and postnatal rodent models specifications table. To address this goal, the treadmill was developed, as an interdisciplinary capstone design project largely using off-the-shelf components and open-source technology. This open-source treadmill design offers additional customization for the end user at lower costs. The treadmill not only fits inside a standard neonatal incubator, but also allows user-specified control over speed and incline. A transparent belt and enclosure design allow video recording for gait analysis. Overall, the treadmill is useful for investigating the impacts of locomotor behavior during neonatal and postnatal development.

## 2 Hardware description

**Fig. 1:**
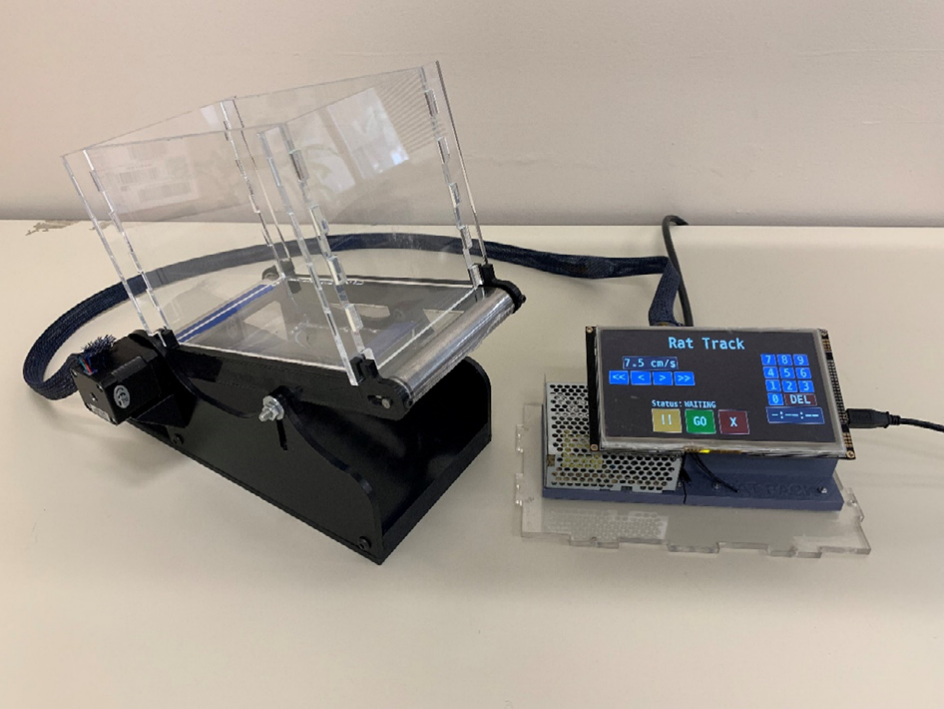
Treadmill with acrylic containment walls, and touchscreen control device.

The treadmill (Fig. 1) consists of a transparent moving belt elevated above a platform where a motion tracking device or camera could be placed. The belt surface can be inclined between 0 and 15 degrees and is driven by a stepper motor that can operate at speeds between 0.5 and 15 cm/s. Above the belt surface a removable and transparent acrylic enclosure contains the subject and allows video recording and gait tracking from the sides and front. An external control box houses the majority of the electronics including an intuitive touchscreen interface. This interface is used for setting the belt speed and run time. Furthermore, the control box is separate from the treadmill; this allows the treadmill to be controlled from outside of a neonatal incubator or another type of environmental chamber.

• Simple, compact design using widely available components for easy replication
• Designed for use with smaller and younger rodent models
• Low overall cost with speed and incline control
• Transparent design facilitates a variety of video analysis methods

## 3 Table design files summary

### 3.1 3D printed parts

**Fig. 2:**
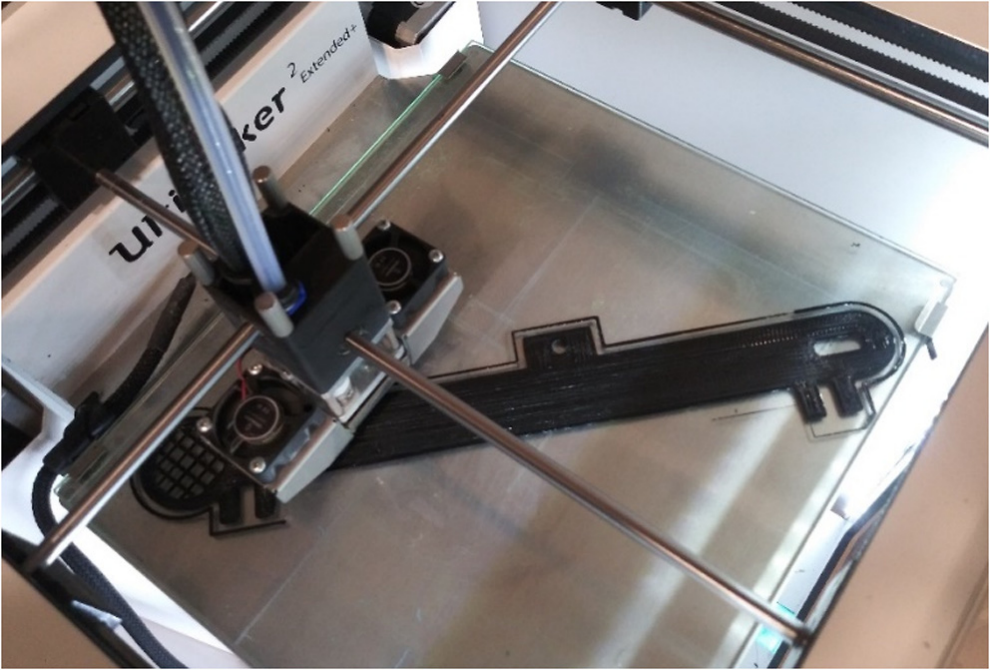
3D printing the support arms.

Support structures for the stepper motor, power supply, and Arduino were 3D printed on a Sindoh 3DWOX (Seoul, Korea) using proprietary Sindoh 3DWox filament. Belt arms were completed using polyethylene terephthalate (PETG) filament and support structures with acrylonitrile butadiene styrene (ABS) (Fig. 2). While a Sindoh 3D printer was used here, most 3D printers with PLA (Polylactic Acid) or ABS material would work as well.

### 3.2 Machined parts

**Fig. 3:**
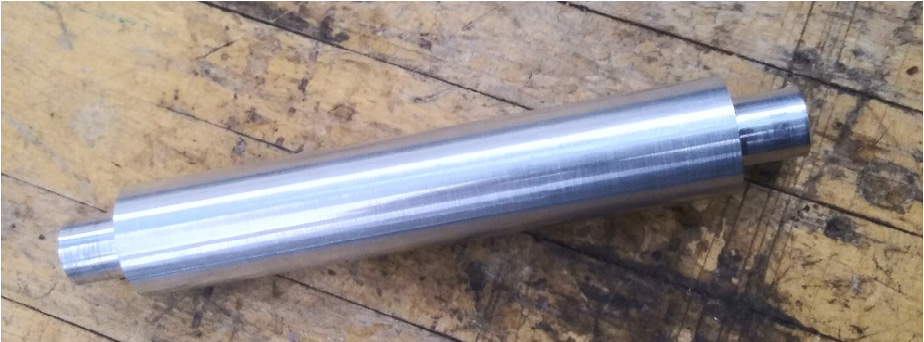
Machined drive roller, cut on a CNC lathe.

A custom drive roller that is connected to the motor was machined from a single piece of aluminum stock (Fig. 3).

### 3.3 Laser cut parts

**Fig. 4:**
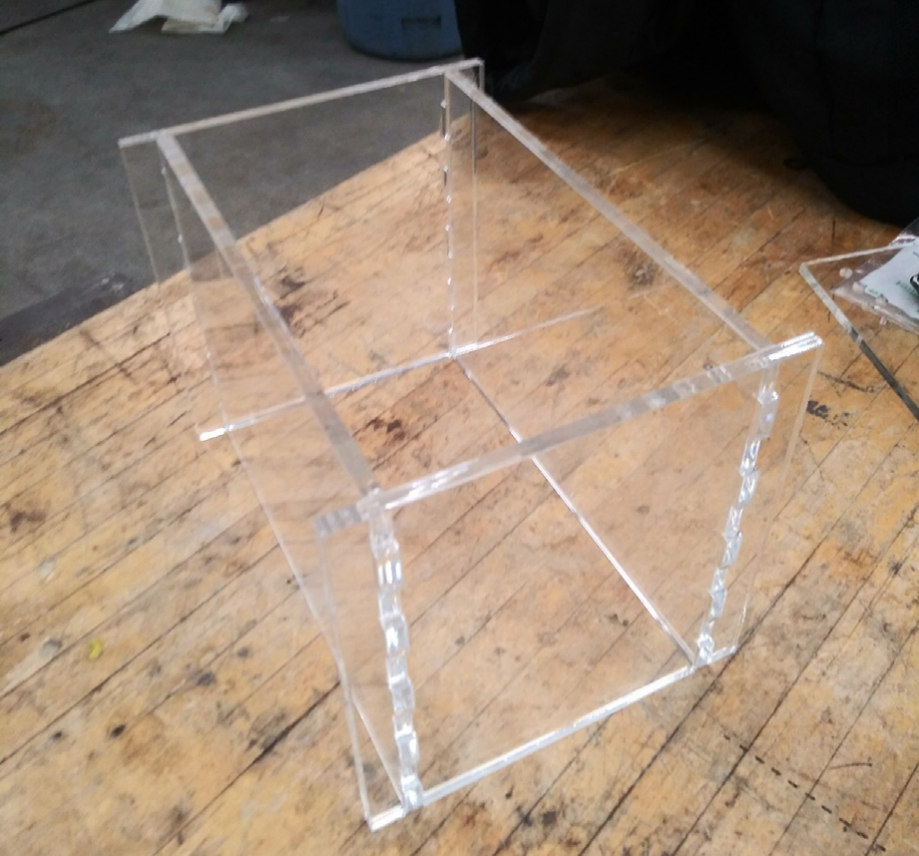
Acrylic containment walls after being epoxied together.

The treadmill belt was laser cut out of clear polyvinylchloride (PVC) before being glued into a continuous loop with epoxy. The containment enclosure walls (Fig. 4), treadmill base sides, and control box walls were all laser cut from 1/4″ thick acrylic sheet.

### 3.4 Electronics

**Fig. 5:**
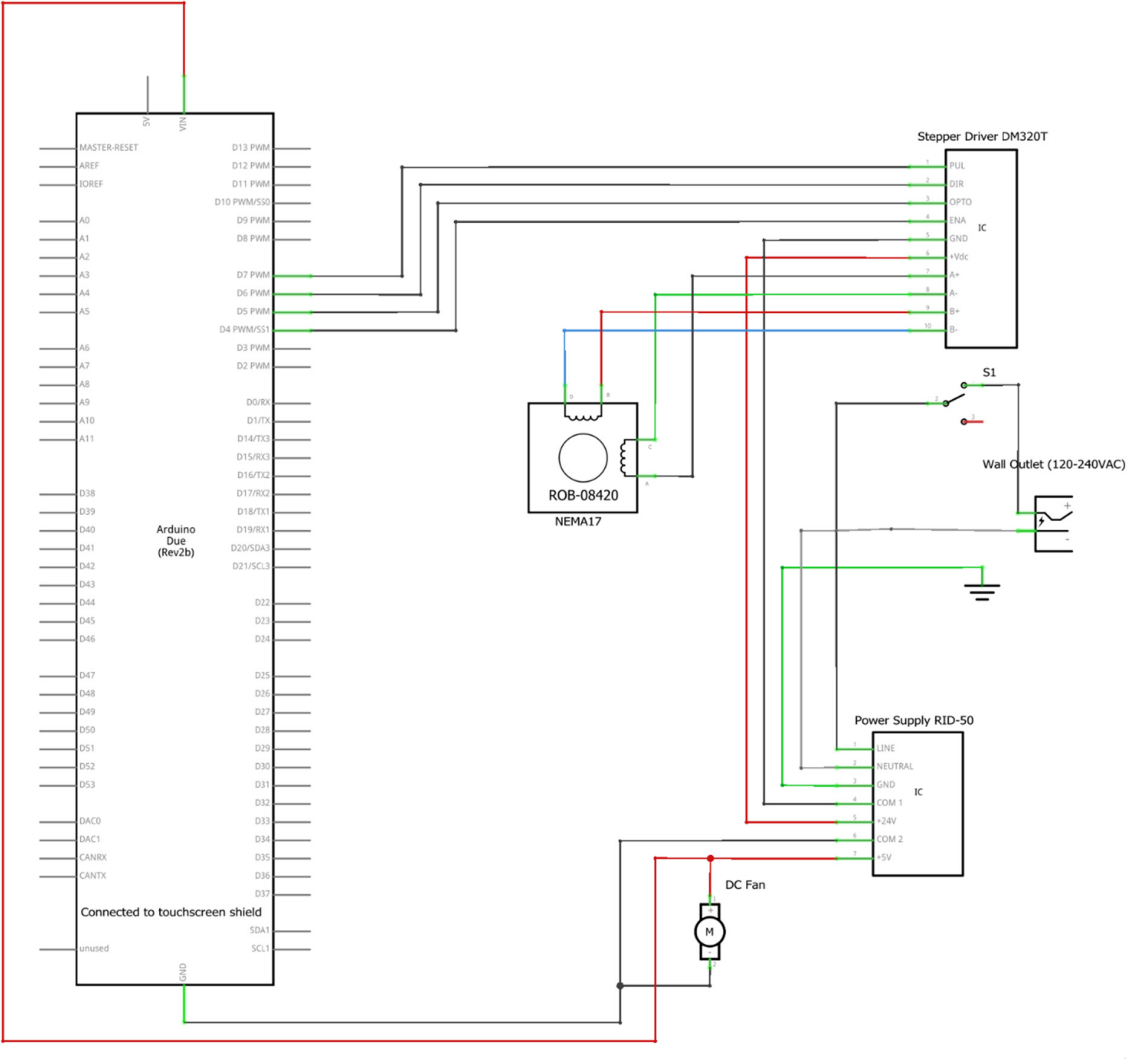
Circuit schematic of control enclosure.

The schematic diagram for the electronic circuitry is shown in Fig. 5. Components include an Arduino DUE, 7″ TFT touchscreen/shield, DM320T stepper driver, and a NEMA 17 stepper motor. Power inputs for the motor was 18–30 V and the Arduino DUE was 5 V, thus a duel voltage 24VDC/5VDC power supply was used.

### 3.5 Software

All code was written in Arduino’s built-in language and requires Arduino IDE for code execution.

**Design Files Summary:** 

Design file name	File type	Open source license	Location of the file
PrintedSupport2STL	STL	GNU General Public License v3	https://osf.io/5bscv/?view_only=02ceddd2b43848c5aeaa8e9c519691db
PrintedSupportSTL	STL	GNU General Public License v3	https://osf.io/krqhx/?view_only=02ceddd2b43848c5aeaa8e9c519691db
Support-ArduinoSTL	STL	GNU General Public License v3	https://osf.io/h4gj3/?view_only=02ceddd2b43848c5aeaa8e9c519691db
Motor Support	STL	GNU General Public License v3	https://osf.io/tfq73/?view_only=02ceddd2b43848c5aeaa8e9c519691db
Upper frame right	STL	GNU General Public License v3	https://osf.io/85u2s/?view_only=02ceddd2b43848c5aeaa8e9c519691db
Upper frame left	STL	GNU General Public License v3	https://osf.io/a8kx6/?view_only=02ceddd2b43848c5aeaa8e9c519691db
Driver Roller	SLDPRT	GNU General Public License v3	https://osf.io/usmxp/?view_only=02ceddd2b43848c5aeaa8e9c519691db
Outside Frame L	DXF	GNU General Public License v3	https://osf.io/te5bn/?view_only=02ceddd2b43848c5aeaa8e9c519691db
Outside Frame R	DXF	GNU General Public License v3	https://osf.io/5cb9n/?view_only=02ceddd2b43848c5aeaa8e9c519691db
Frontboxwall	DXF	GNU General Public License v3	https://osf.io/2pcng/?view_only=02ceddd2b43848c5aeaa8e9c519691db
Sideboxwall	DXF	GNU General Public License v3	https://osf.io/q4ysj/?view_only=02ceddd2b43848c5aeaa8e9c519691db
ControlRightSideWall	DXF	GNU General Public License v3	https://osf.io/jqac9/?view_only=02ceddd2b43848c5aeaa8e9c519691db
ControlLeftSideWall	DXF	GNU General Public License v3	https://osf.io/3htbz/?view_only=02ceddd2b43848c5aeaa8e9c519691db
ControlFrontWall	DXF	GNU General Public License v3	https://osf.io/tuza7/?view_only=02ceddd2b43848c5aeaa8e9c519691db
ControlBackWall	DXF	GNU General Public License v3	https://osf.io/vqmej/?view_only=02ceddd2b43848c5aeaa8e9c519691db
ControlTopWall	DXF	GNU General Public License v3	https://osf.io/w8mge/?view_only=02ceddd2b43848c5aeaa8e9c519691db
ControlBottomWall	DXF	GNU General Public License v3	https://osf.io/k5g38/?view_only=02ceddd2b43848c5aeaa8e9c519691db
RatTrack_FinalCode	ino	GNU General Public License v3	https://osf.io/ujm3h/?view_only=02ceddd2b43848c5aeaa8e9c519691db

• PrintedSupport2STL – 3D printed base for the power supply. Bolts into ControlBottomWall
• PrintedSupportSTL – 3D printed base for the Arduino DUE/Shield. Bolts into ControlBottomWall
• Support-ArduinoSTL – 3D printed support for the Touchscreen. Attaches to Arduino shield.
• Motor Mount − 3D printed support for the motor. Attaches to the right outer frame.
• Upper frame right – 3D printed arm piece supporting the rollers and allows for angle adjustment.
• Upper frame left – 3D printed arm piece supporting the rollers and allows for angle adjustment.
• Driver Roller – CNC-turned roller with a crown to center the belt and attaches to the motor shaft via a set-screw.
• Treadmill Base Sidewall L – Easily detachable frame that supports the left belt arm and angle adjustment.
• Base Sidewall R – Frame that has the motor attached to the rear and performs the same support functions for the right belt arm and angle adjustment.
• Sideboxwall – Side walls for the containment structure that surrounds the test animal on the treadmill. Laser cut.
• Frontboxwall – Front wall and back walls for the containment structure that surrounds the test animal on the treadmill. Laser cut.
• ControlRightSideWall – Right wall of control enclosure. Laser cut.
• ControlLeftSideWall – Left wall of control enclosure. Laser cut. Holds DC fan and toggle switch.
• ControlFrontWall – Front wall of control enclosure. Laser cut. One edge beveled.
• ControlBackWall – Back wall of control enclosure. Laser cut. One edge beveled. Contains cutout for power cord cable gland and female EPS cable.
• ControlTopWall – Top wall of control enclosure. Laser cut. Two ends beveled. Covers touchscreen edges.
• ControlBottomWall – Bottom wall of control enclosure. Holds PrintedSupport and PrintedSupport2.
• RatTrack_FinalCode – Arduino code for the DUE and touchscreen.

## 4 Table bill of materials

**Bill of Materials:** 

Designator	Component	Number	Cost per unit – currency	Total cost – currency	Source of materials	Material type
Power Supply	RID-50B	1	24.86 USD	24.86 USD	https://www.digikey.com/product-detail/en/mean-well-usa-inc/RID-50B/1866-3997-ND/7705998	Semi-conductor, metal
AC Power Cord	Power Cord, SJT 16AWG 1.5m	1	4.76 USD	4.76 USD	https://www.digikey.com/product-detail/en/AK500%2f16-OE-5-1.5/AE10675-ND/2504516/?itemSeq=288091111	Metal, Polymer
Cable Gland	PG 11 Cable Gland	1	1.63 USD	1.63 USD	https://www.digikey.com/product-detail/en/amphenol-industrial-operations/AIO-CSPG11/AIO-CSPG11-ND/3904978	Polymer
Wire supports	C-Type cable clip	2	0.51	1.02 USD	https://www.digikey.com/product-detail/en/KKD-4-RT/RPC1093-ND/391921/?itemSeq=288091148	Polymer
DC Fan	Axial 5VDC Fan	1	8.23 USD	8.23 USD	https://www.digikey.com/product-detail/en/F410T-05MC/563-1132-ND/1165526/?itemSeq=288091815	Polymer, metal
Toggle Switch	125V 20A Rocker Switch	1	5.51 USD	5.51 USD	https://www.digikey.com/product-detail/en/1-1571095-1/450-1666-ND/1755860/?itemSeq=288092178	Polymer, metal
Arduino DUE	Arduino DUE + Plastic Base	1	38.50 USD	38.50 USD	https://store.arduino.cc/usa/due	Semi-conductor, polymer, metal
Touchscreen+Shield	7″ Resistive Touch Screen Shield w/Touch Panel	1	43.32 USD	43.32 USD	https://www.buydisplay.com/default/7-inch-arduino-touch-screen-shield-ssd1963-library-for-mega-due	Semi-conductor, polymer
Stepper Motor	NEMA 17 Stepper Motor 59Ncm	1	13.99 USD	13.99 USD	https://www.amazon.com/Stepper-Bipolar-Connector-compatible-Printer/dp/B00PNEQKC0/ref=sr_1_16?keywords=Pololu+A4988&qid=1551752141&s=gateway&sr=8-16	Metal, semi-conductor
Stepper Driver	DM320T Stepper Driver	1	22.99 USD	22.99 USD	https://www.amazon.com/STEPPERONLINE-0-3-2-2A-18-30VDC-Micro-step-Resolutions/dp/B075R88FMN/ref=sr_1_3?keywords=stepper+driver+nema+17&qid=1551985449&s=hi&sr=1-3	Metal, semi-conductor
3D Printer Filament	Sindoh 3DWox PLA Filament Refill	1	29.99 USD	29.99 USD	https://www.amazon.com/Sindoh-Filament-Compatible-millimeters-Diameter/dp/B01HD9ABCW/ref=sr_1_1?keywords=3dwox+pla&qid=1556766202&s=gateway&sr=8-1	Polymer
Arduino DUE power/Coding cable	90 Degree Micro-USB to USB-2.0	1	7.99 USD	7.99 USD	https://www.amazon.com/RAYSUN-Packs-Degree-Micro-Male/dp/B00WMF7JUA/ref=sr_1_5?keywords=right+angle+usb+micro&qid=1556766578&s=gateway&sr=8-5	Polymer, metal
Male/Female Wires	Male/Female Breadboard Jumper Wires	1	5.79 USD	5.79 USD	https://www.amazon.com/EDGELEC-Breadboard-Optional-Assorted-Multicolored/dp/B07GD2BWPY/ref=sr_1_1_sspa?keywords=female+to+male+wires&qid=1556769900&s=gateway&sr=8-1-spons&psc=1	Polymer, metal
Right Angle Pin Headers	Right Angle Connector	1	1.07 USD	1.07 USD	https://www.digikey.com/product-detail/en/sullins-connector-solutions/PBC12SBAN/S1111E-12-ND/860352	Polymer, metal
EPS Cable	8pin EPS Power Extension Cable	1	4.98 USD	4.98 USD	https://www.amazon.com/8in-EPS-Power-Extension-Cable/dp/B000M802RG/ref=sr_1_1_sspa?keywords=8pin+cpu+cable&qid=1556768920&s=gateway&sr=8-1-spons&psc=1	Polymer, metal
Clear vinyl belt material	CLEAR MARINE VINYL – FLEXA®, 20 gauge	1	6.75 USD/ yard	6.75 USD	https://www.marinevinylfabric.com/products/clear-marine-vinyl?variant=14023822049364&gclid=CjwKCAjwza_mBRBTEiwASDWVvgdS4FJH7yziSpOg90xvXsJvpJ-tusI3KtUpoINYdnEFi1rz5sKrBxoCYnsQAvD_BwE	Polymer
1/4″ Acrylic stock (0.23” actual)		1@ 24″x48″x1/4″	62.45 USD	62.45 USD	https://www.mcmaster.com/8589K84	Polymer
Front Roller	7/8″ Diameter Steel Conveyor Roller for 4–7/8″ Between Frame Width	1	8.65 USD	8.65 USD	https://www.mcmaster.com/2287T11	Metal
Aluminum stock for driving roller	Tight-Tolerance 6061 Aluminum Rod	1 @ 1″ Diameter, 1ft length	18.27 USD	18.27 USD	https://www.mcmaster.com/9062K21	Metal
Roller ball bearings	R8 for ½″ shaft ball bearings	2	6.27 USD	12.54 USD	https://www.mcmaster.com/60355K505	Metal
Plastic base	Black UHMW Sheet	1@ 6″x12″x1″	28.06 USD	28.06 USD	https://www.mcmaster.com/4296A182	Polymer
Clear epoxy adhesive			10.89 USD	10.89 USD		
Tensioner bolts	6–32 × ¾″ SHCS	2	0.16 USD	0.32 USD		Metal
Motor mount bolts	8–32 × 5/8″ SHCS	2	0.17 USD	0.34 USD		Metal
Nuts	8–32 Nuts	2	0.05 USD	0.10 USD		Metal
Right side base bolts	¼–20 × ¾ SHCS	2	0.87 USD	1.74 USD		Metal
Studs	¼-20 × 2″ Threaded bars	2	0.50 USD	1.00 USD		Metal
Threaded inserts	¼–20 female, 7/16–14 male, 0.437″ length	4	4.10 USD	16.40 USD		Metal
Angle adjustment hex head bolt	¼–20 × ¾″	2	1.90 USD	3.80 USD		Metal
Nuts	¼–20	2	0.20 USD	0.40 USD		Metal
Wingnuts	¼–20	2	0.95 USD	1.90 USD		Metal
Washers	¼	4	0.11 USD	0.44 USD		Metal
Set screw	10–32 Set screw	1	0.46 USD	0.46 USD		Metal
M3 × 0.50 × 8 Bolts		2	0.35 USD	0.70 USD		Metal
M3 × 0.50 × 10		12	0.43 USD	5.16 USD		Metal
M3 × 0.50 × 12 Bolts		2	0.47 USD	0.94 USD		Metal
M3 × 0.50 × 16 Bolts		6	0.55 USD	3.30 USD		Metal
M3 Nuts		6	0.33 USD	1.98 USD		Metal
M3 Washers		4	0.09 USD	0.36 USD		Metal
M2 × 8 pan machine screws		2	0.33 USD	0.66 USD		Metal
M2 Washers		2	0.23 USD	0.46 USD		Metal
#4 × ½ pan sheet metal screws		4	0.09 USD	0.36 USD		Metal
#6 × ½ pan sheet metal screws		2	0.10 USD	0.20 USD		Metal

## 5 Build instructions

### 5.1 Treadmill construction

• Cut a groove on the base frame of the treadmill as per the drawings instructions using an end mill.
• Drill two holes on each side to a depth compliant with the attachment bolt of your preference for the side walls.
• Tap each of the four holes with the correct sized tap pertaining to the hole size you just drilled.
• Insert threaded inserts into the holes with Loctite® threadlocker coating on the outside.
• Laser cut OuterFrameLeft and OuterFrameRight and two of each of the SideWall and FrontWall box pieces, all from 1/4″ clear acrylic
• Turn down the back roller from aluminum stock using a CNC lathe
• 3D print UpperArmRight, UpperArmLeft, and Motor Mount using an infill density of at least 25%.
• Laser cut the belt from clear ¼ inch (20-gauge) plastic vinyl to the specifications listed in the drawing packages.
• Mill out an appropriate through-hole on each side of purchased front roller.

### 5.2 Treadmill assembly

• First thread two **¼–20 × 2″ threaded rods** into the **left** side threaded inserts of the base frame.
• Attach OuterFrameRight to the right side of the base frame.
• Secure with two **¼–20 × ¾″** **bolts** threaded into the inserts.
• Insert the two bearings into each of the upper frame arm cavities.
• Screw the motor mount to the outer right frame wall with the flat of the motor mount towards the top using two **8–32 × 1″ bolts**.
• Slide the motor onto the motor mount and insert the shaft through the outer frame wall.
• Attach the motor with four **M3 × 10 bolts** to the outer frame wall.
• Hold UpperArmRight in place with the motor shaft through the middle of the bearing hole.
• Slide on the back roller through the bearing hole and secure the roller to the motor shaft with the 10–32 set screw.
• Run the front roller through the right upper arm slot and thread the **6–32 × 5/8″ bolts** through the hole in the front roller to hold it in place.
• Slide on the belt.
• Attach UpperArmLeft to the end of the back roller through the bearing hole and slide the front roller into the slot on the end of the upper arm.
• Attach the second tensioning screw to the front roller on the left side.
• Slide the outer frame left piece onto the threaded rods and push till flush with the base frame exterior.
• Secure the outer left wall with two **¼ × 20 wingnuts**.
• Run two **¼–20 × 1″ bolts** through the angle adjustment attachment holes on each of the upper arms.
• Secure in place with a nut and appropriate washers.
• Assemble two side walls and front pieces for the upper enclosure box.
• Attach box to the treadmill with the alignment tabs on top of the upper arms.

### 5.3 Control enclosure construction

**Fig. 6:**
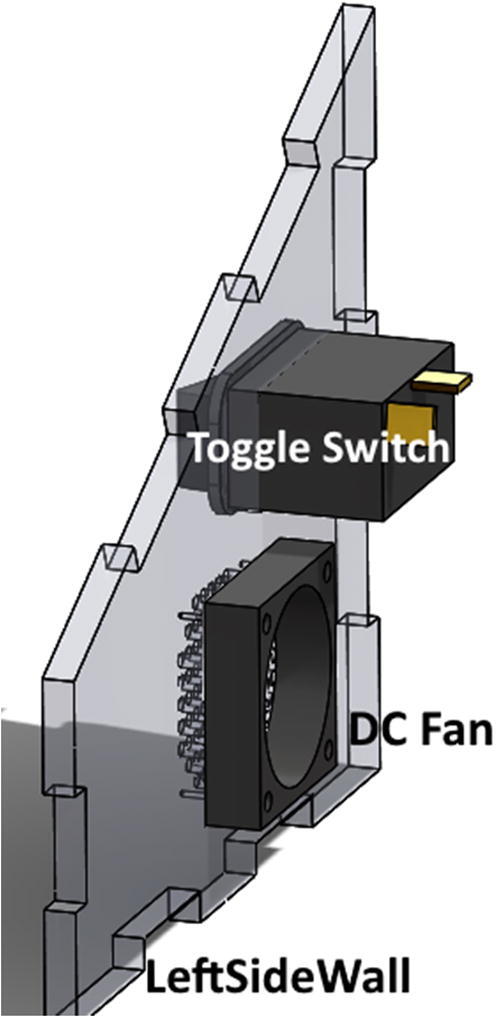
Placement of DC fan and toggle switch on ControlLeftSideWall.
• Using a Dremel, mill, file, or any capable tool, machine out designated clearance in ControlTopWall for Touchscreen pins (A mill is recommended, but a Dremel is good option). Clearance does not have to follow specified dimensions exactly (Fig. 7), just enough so ControlTopWall lays flush over Touchscreen.

• 3D print PrintedSupportSTL, PrintedSupport2STL, and Support-ArduinoSTL using recommended settings and a nozzle diameter of 0.4 mm or smaller.
• Connect Arduino DUE to computer using Micro-USB cable.
• Upload RatTrack_FinalCode to Arduino DUE, making sure to set proper Board Type within Arduino IDE software. (https://www.arduino.cc/en/Guide/ArduinoDue)
• Cut ControlRightSideWall, ControlLeftSideWall, ControlFrontWall, ControlBackWall, ControlTopWall, and ControlBottomWall out of acrylic using a laser cutter, water jet, or any other CNC-capable machine using DXF files.
• Counterbore ControlBottomWall holes on bottom surface using #1 size drill so **M3 bolts** heads lay below surface. See ControlBottomWall PDF drawing for details.
• Pre-drill DC Fan holes in ControlLeftSideWall so screws will not split acrylic.
• Screw DC Fan into inside face of ControlLeftSideWall using four **#4 × ½ pan-head sheet metal screws**.
• Press toggle-switch through outside face of ControlLeftSideWall (Fig. 6)

**Fig. 7:**
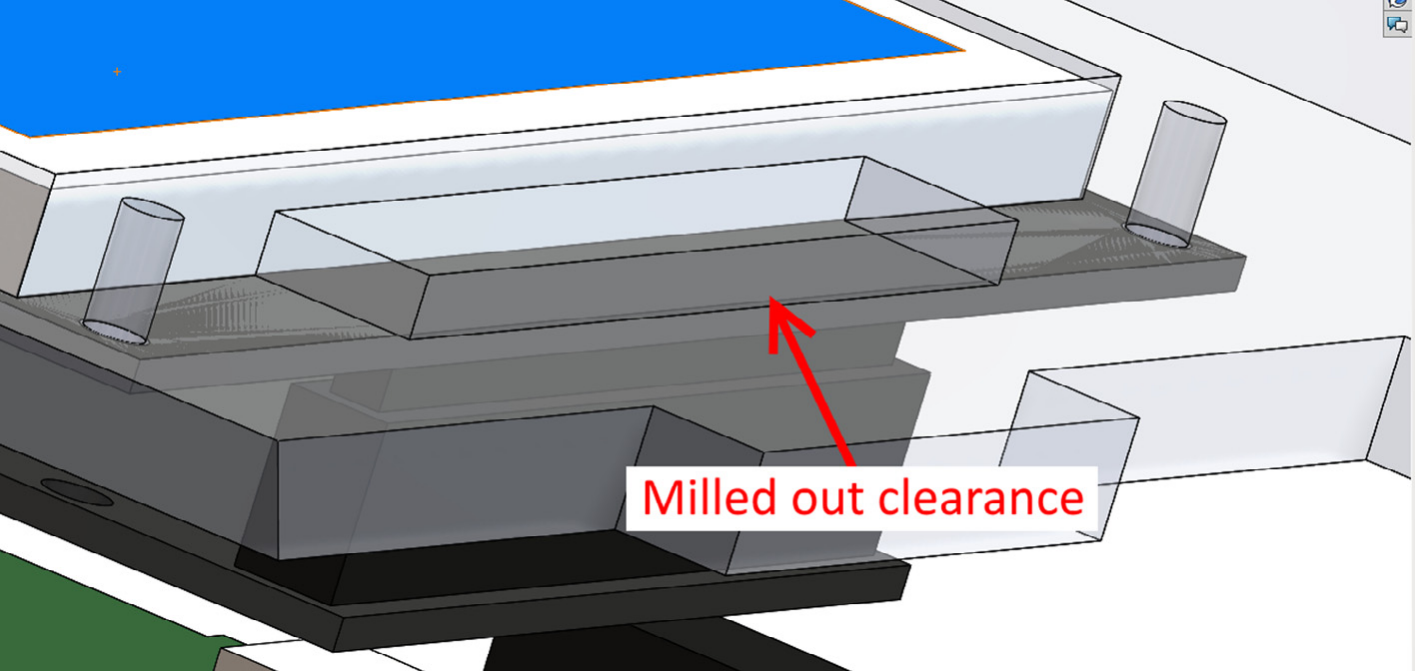
Clearance for touchscreen pins (milled out from bottom side of TopWall).
• Using a table saw, file, or angled jig on a laser cutter (Fig. 8), machine the bevels on ControlFrontWall, ControlTopWall, and ControlBackWall. See Fig. 9 and drawing files for clarification on angles.

**Fig. 8:**
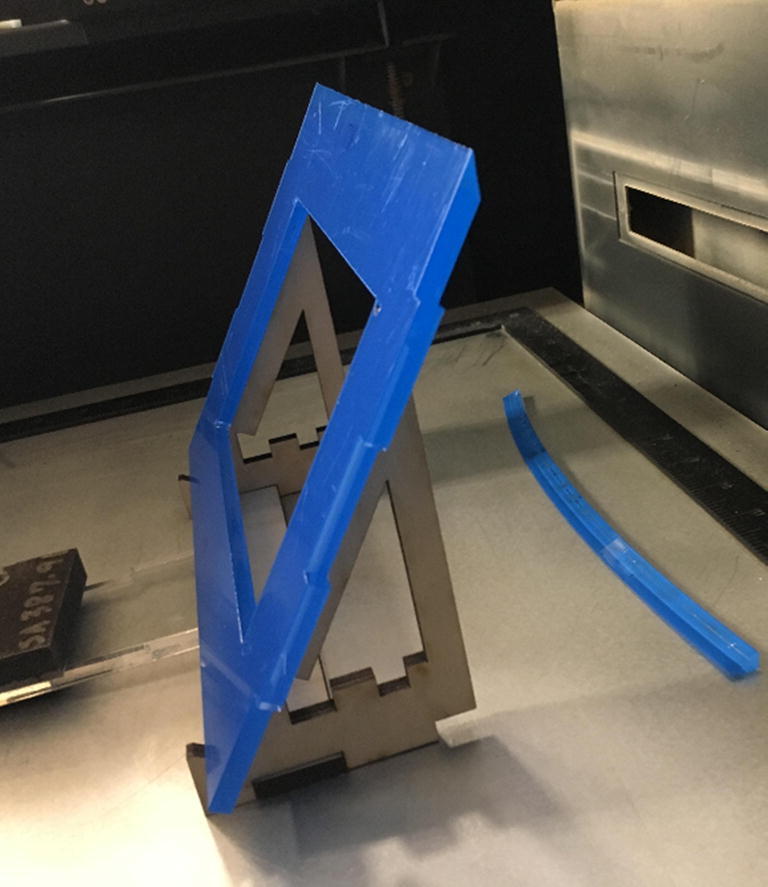
Example of an angled jig for laser cutter.

**Fig. 9:**
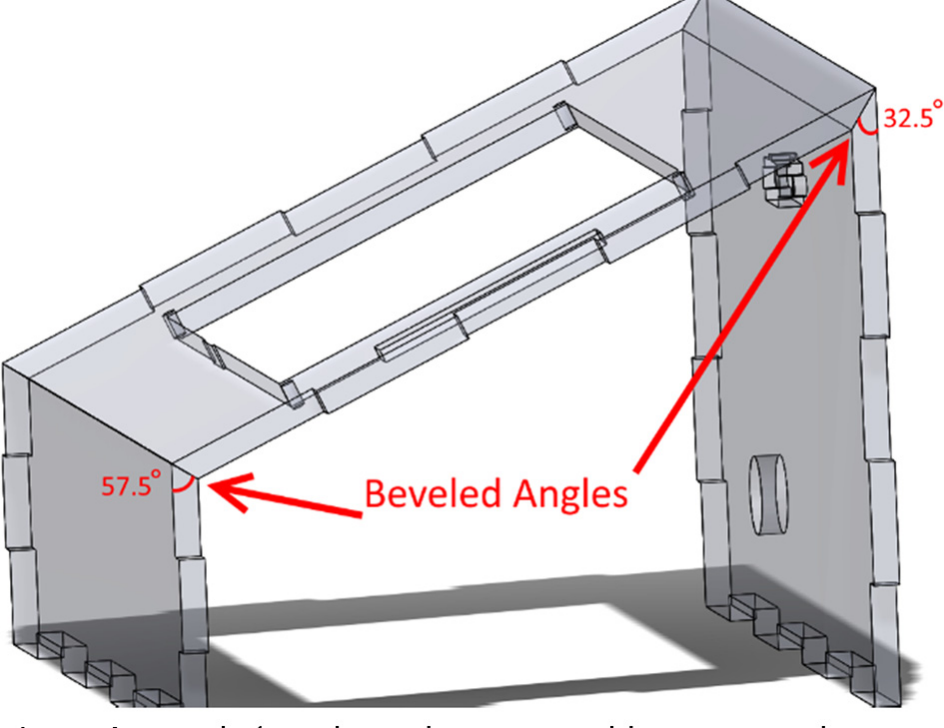
Beveled angles to be cut on table-saw, or other means.
• Mill out clearance for female EPS cable wing-clips from outside face of ControlBackWall. See ControlBackWall drawing for details. Ensure wing-clips will snap outwards when female end is pressed through.
• Glue the four side walls together with epoxy. Position on ControlBottomWall to ensure side walls are properly aligned. Secure with masking tape temporarily to hold in place. **Do NOT epoxy ControlBottomWall or ControlTopWall to side walls.**
• After side walls have dried, predrill 4 outside holes on ControlBottomWall (2 on Left Side, 2 on Right Side) through the side walls. This will ensure that securing bolts will not split through acrylic wall edges.
• Place clear-plastic Arduino base on PrintedSupport. Secure Arduino to clear plastic base, and clear plastic base to PrintedSupport with designated bolts. Fig. 10 shows the placement of PrintedSupport, Arduino/Shield, and Support-Arduino

**Fig. 10:**
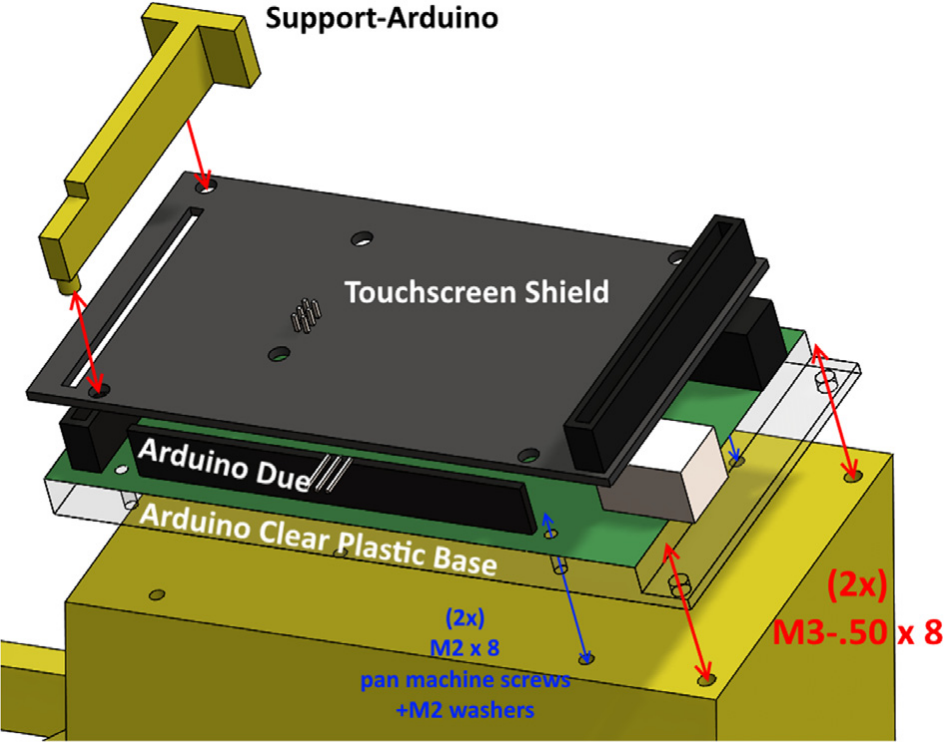
Bolts to secure Arduino plastic base + DUE board/Shield, along with placement of Support-Arduino, which will support the touchscreen once plugged into Shield.
• Secure DM320T Stepper Driver to PrintedSupport using two **#6 × ½″** **sheet metal screws** (Fig. 11).

**Fig. 11:**
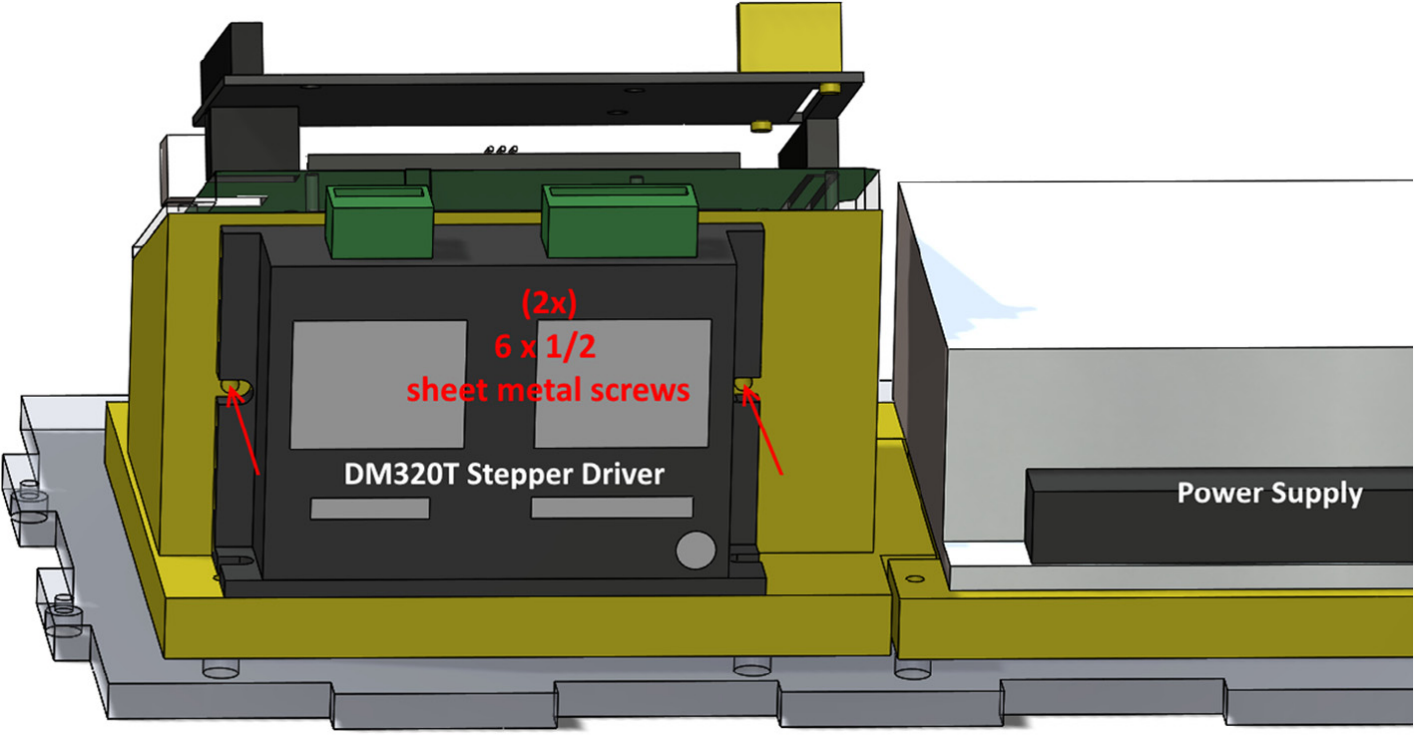
Screws to secure Stepper Driver to PrintedSupport.
• Secure PrintedSupport and PrintedSupport2 with six **M3 × 0.50 × 16 bolts**. Secure with **M3 nuts** where possible (the two bolts at DM320T base may be difficult to secure with nuts. Nuts not necessary in these two spots). Secure Power Supply to Printed Support2 using two **M3 × .50** **×** **12 bolts** (power supply bolts are shorter to avoid intrusion into circuitry) (Fig. 12).

**Fig. 12:**
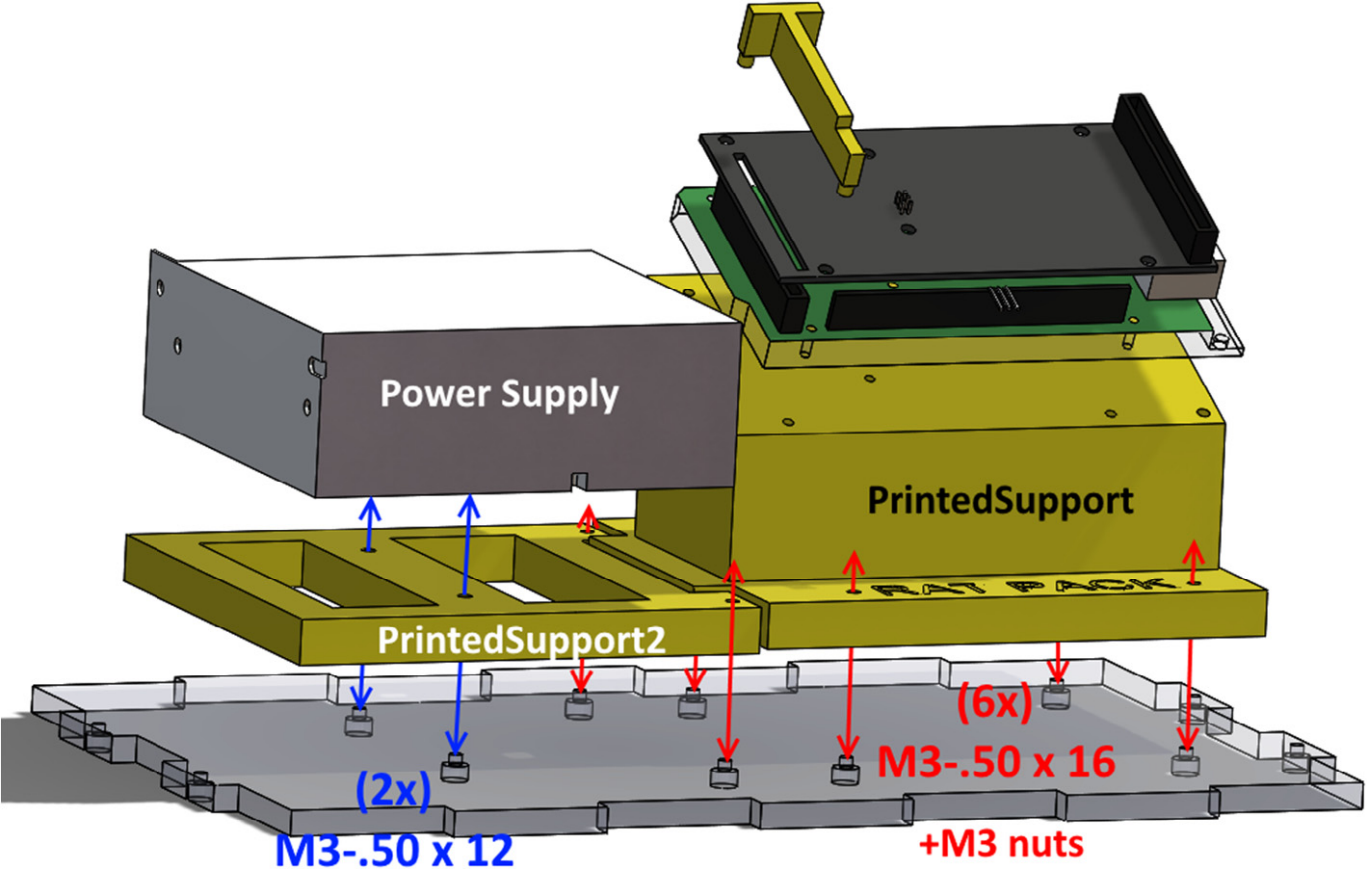
Placement and bolts to secure Power Supply, Printed Supports, and Arduino/Shield assembly.

### 5.4 Wiring

**Fig. 13:**
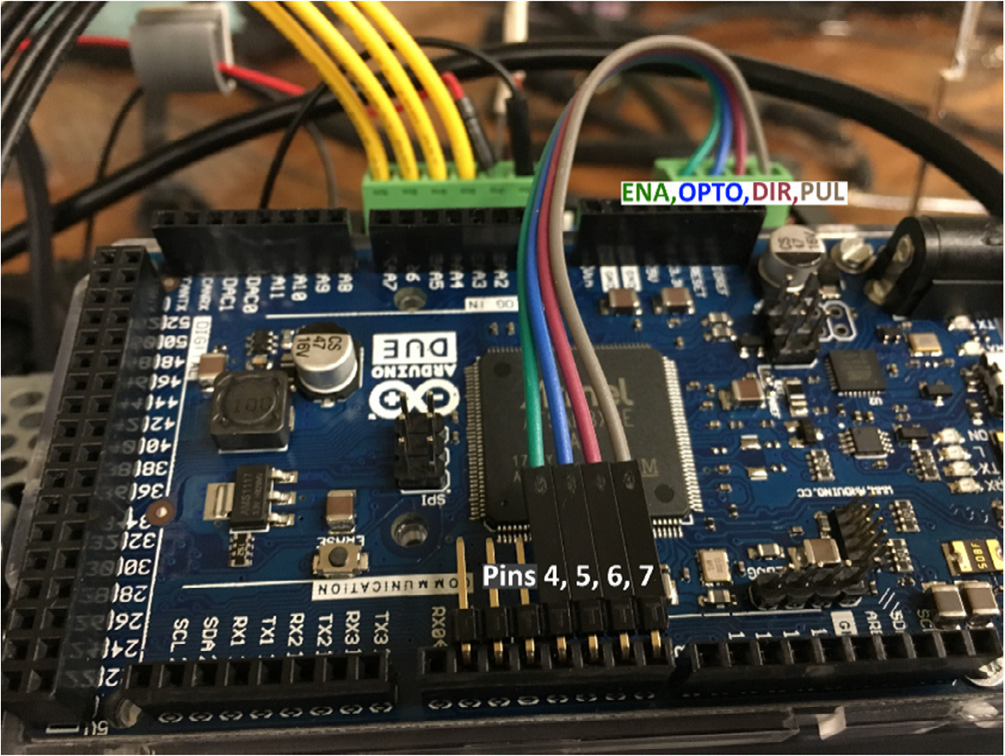
Jumper wires from DM320T terminals connect to right angle pin header into Arduino DUE.
• Attach cable gland to outside of ControlBackWall, securing with attached nylon nut on inside face (Fig. 14).

• Attach 4 male/female jumper wires to PUL, DIR, OPTO, ENA of DM320T Stepper Driver to Arduino DUE digital pins 7, 6, 5, 4 respectively using right angle pin headers (Fig. 13).

**Fig. 14:**
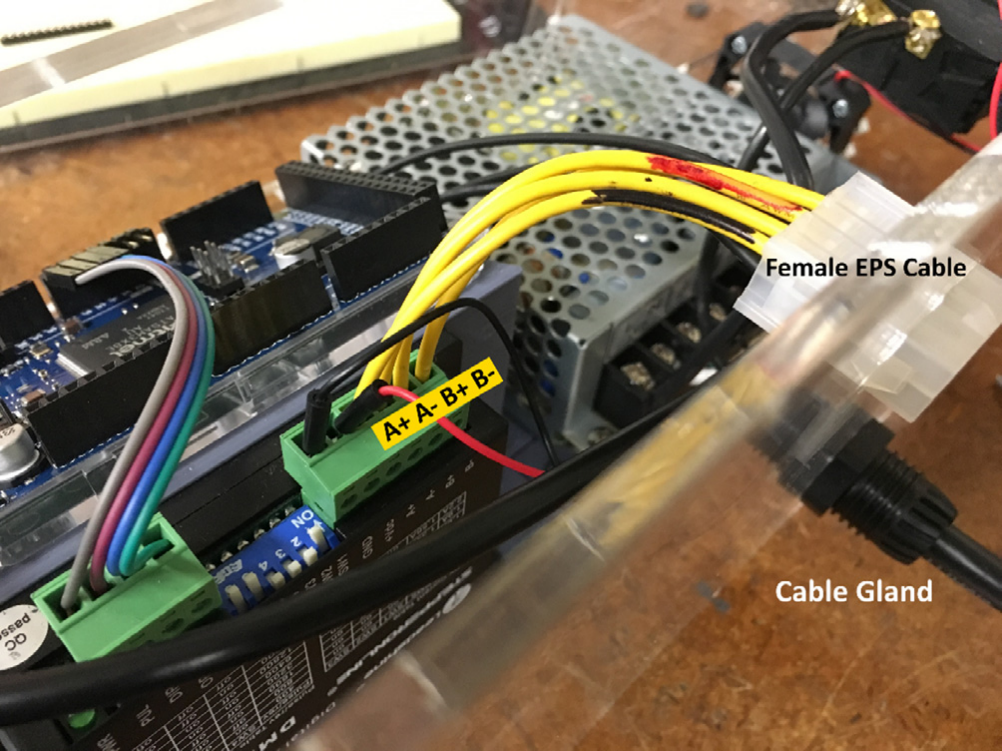
Female EPS cable wires connect to DM320T motor terminals. Cable gland inserted through outside face of ControlBackWall.
• Split EPS cable in half. Strip off about ¼″ from ends of 4 wires and attach to A+, A-, B+, B- terminal ports of DM320T Stepper Driver (Fig. 14).
• Cut off female connector from end of NEMA 17 Stepper Motor. Solder the four wires to the **male** end of the EPS cable, such that the BLACK wire connects to A+, the GREEN wire to A-, the RED wire to B+, and the BLUE wire to B-. See the circuit schematic (Fig. 5) for further clarification.
• Plug Micro-USB cord Arduino DUE port. (Attached micro-USB cord should angle towards DM320T and backwall to connect to power supply. Choose correct facing USB cord).
• Cut off USB 2.0 end from right-angled Micro-USB cord. Strip ends of Micro-USB cord to expose BLACK and RED wires. Secure BLACK to COM1 and RED to + V1. Retain the other Micro-USB/USB 2.0 cord for future use recoding Arduino DUE.
• Attach jumper wires from + V2 and COM2 to + Vdc and GND terminals of DM320T, respectively.
• Strip off about ¼″ from three ends of AC power cord.
• Feed all three wires through cable gland.
• Cut off roughly 3″ off WHITE and GREEN wires of power cord to shorten wires to fit to power supply. Leave BLACK wire long.
• Attach WHITE wire to N (neutral), and GREEN wire to GND (ground) terminals of power supply.
• Place four side walls onto ControlBottomWall.
• Attach BLACK wire from AC power cord to one terminal of toggle-switch.
• Use cut off, or spare 16AWG wire to attach other terminal of toggle-switch to L (line) terminal of power supply.
• Attach RED and BLACK DC Fan wires to + V1 and COM1 respectively.
• Press **female** EPS cable through inside of ControlBackWall, making sure the wing-clips snap outwards (Fig. 15).

**Fig. 15:**
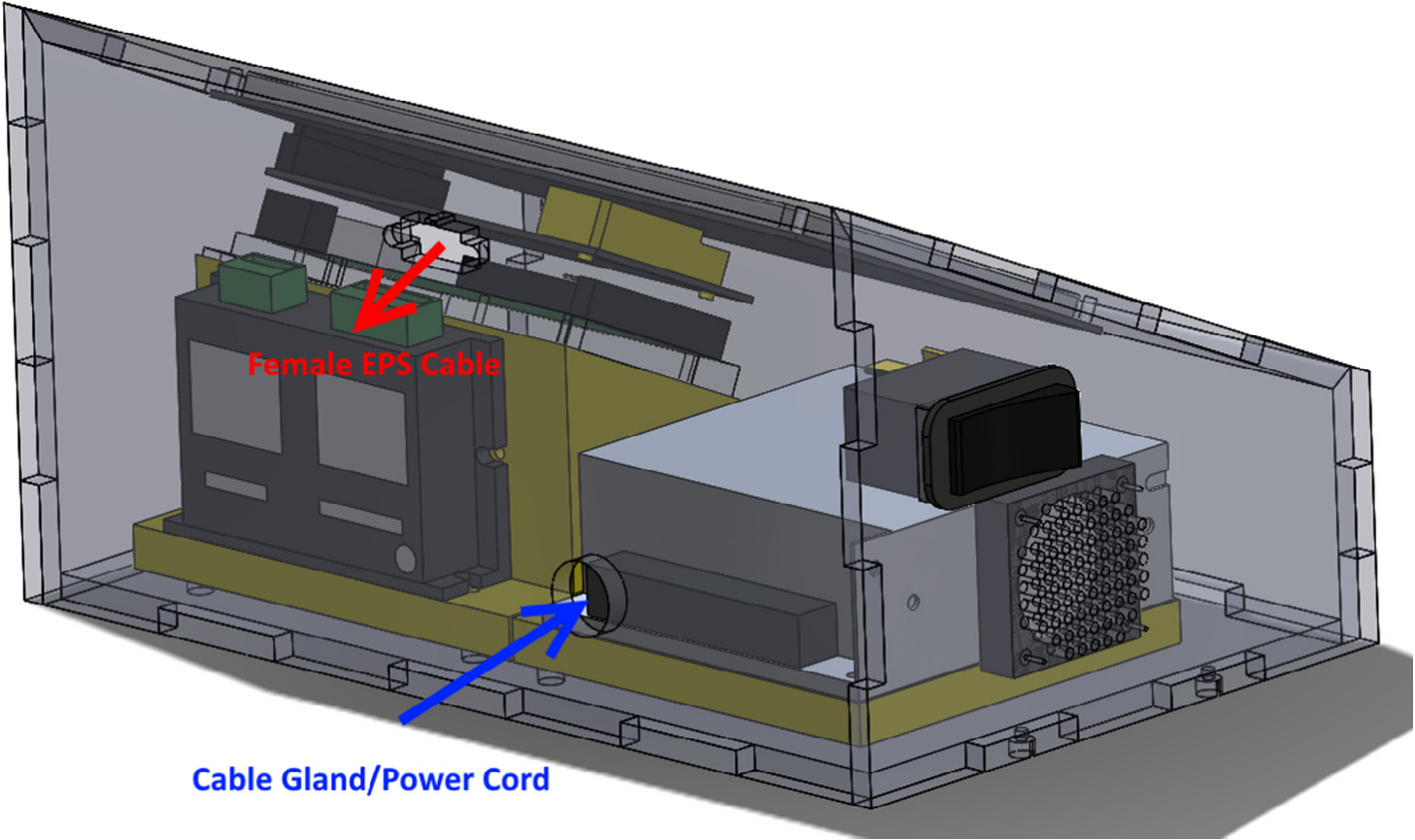
Location of Cable Gland and Female EPS cable placement.
• Neatly route wires using adhesive wire support clips (optional).
• Secure ControlBottomWall to four side walls using four **M3 × 0.50 × 10 bolts**.
• Place ControlTopWall over Touchscreen, secure using four **M3 × 0.50 × 10 bolts and M3 washers** (Fig. 16).

**Fig. 16:**
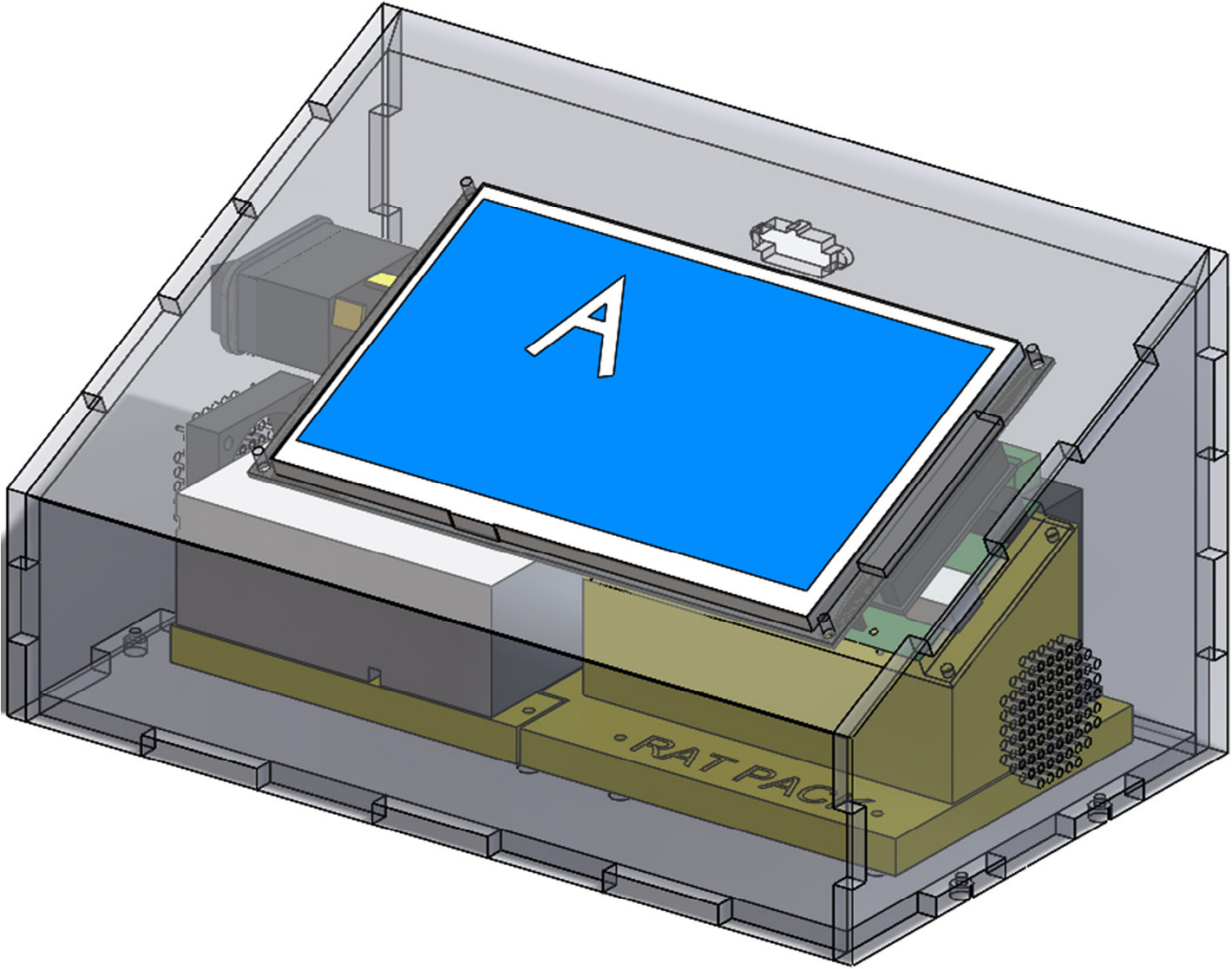
Fully assembled control enclosure.
• Plug in Stepper Motor **male** EPS plug to **female** EPS plug.
• Plug in AC power cord to wall outlet.
• Switch toggle switch to ON position to power device.

## 6 Operation instructions

### 6.1 Touchscreen operation

**Fig. 17:**
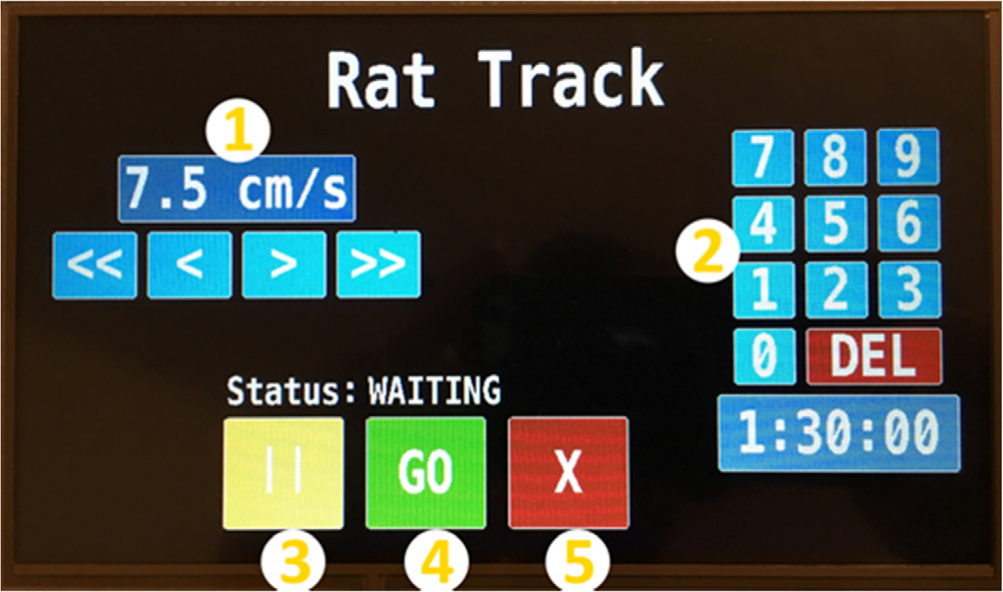
Touchscreen user interface.
• Set the desired duration of the trial in hours, minutes, and seconds by using the number pad **(2)**. DEL key will clear the time. If no time is input, timer will default to 1 h.
• After inputting the desired speed and duration, hit the green GO button **(4)**.
• While running, the speed or time cannot be adjusted.
• A small white timer will count down, located underneath the time duration.
• To pause the trial at any time, press the yellow PAUSE button **(3)**. While paused, the speed or time cannot be adjusted.
• To end the trial early or to adjust speed or duration values, press the red CANCEL button **(5)**.
• Press the red CANCEL button **(5)** at anytime to stop the treadmill.
• When finished, switch the device OFF using the power switch on the left hand side and UNPLUG the AC power cord from the power source (e.g, wall outlet).
• All animal protocols need to be approved by your institution’s animal care and use committee prior to using the device.
• Treadmill running should only occur while under direct supervision.

• Plug in and power on the device using the power switch on left side of control enclosure, wait for the touchscreen to load.
• On the touchscreen (Fig. 17), set the desired speed of the treadmill by using the ≪, <, >, ≫ arrows **(1)**.

### 6.2 Control enclosure disassembly*

*Electric shock hazard

• Ensure the device is switched OFF using the power switch on the left hand side.
• UNPLUG the AC power cord from the power source before disassembly.
• To disassemble enclosure for troubleshooting or maintenance, several M3 hex-pattern bolts need to be removed.
• Loosen 4 bolts on enclosure top wall that connect to the touchscreen. Remove acrylic top.
• Loosen 4 bolts on enclosure bottom wall to loosen side walls.
• With device turned off and unplugged, loosen three AC power cord wires (Black/L, White/N, Green/GND) from the power supply terminals, and the black wire from the toggle-switch on the left wall.
• Loosen two DC fan wires from last power supply 5 V terminals (Red to + V2, Black to COM2)
• Unscrew black cable gland from enclosure back wall to loosen AC power cord.
• Feed AC power cord back out through hole.
• Unplug stepper motor male EPS plugin from back wall.
• Press in on wing-clips on female EPS plugin and press through back wall.
• Lift up to remove enclosure side walls.

### 6.3 Control enclosure reassembly*

*Electric shock hazard

• Before reassembly, ensure the device is switched OFF and the AC power cord is unplugged from the power source.
• To reassemble, replace the enclosure side walls.
• Feed AC power cord through back wall.
• Secure black wires from AC power cord to the toggle-switch, and the other black wire from the toggle-switch to the L terminal of the power supply.
• Secure the white and green wires to the N and GND terminals respectively.
• Tighten black cable gland.
• Reattach two DC fan wires to last power supply terminals (Red to + V2, Black to COM2).
• Press female EPS plugin back through acrylic back wall, making sure the wing-clips snap outwards.
• Tighten 4 bolts on enclosure bottom wall to secure side walls.
• Replace acrylic top over touchscreen. Tighten 4 bolts to secure top wall.
• If acrylic top is too tight or poorly fitting, remove touchscreen from Arduino shield, secure acrylic top to unplugged touchscreen with 4 bolts, then reattach touchscreen pins into Arduino shield.

**Belt Replacement Manual** can be found here:

https://osf.io/p98yd/?view_only=02ceddd2b43848c5aeaa8e9c519691db

**Troubleshooting Guide** can be found here:

https://osf.io/u6pxv/?view_only=02ceddd2b43848c5aeaa8e9c519691db

## 7 Validation and characterization

The treadmill was designed to operate with linear belt speeds of 0.5 to 15 cm/s, with a resolution of 0.1 cm/s. The formula used to control the stepper motor delay based on desired linear velocity was a function of the steps per revolution (controlled by the DM320T Stepper Driver) and the radius of the treadmill’s drive roller, given by:

(1) $$\mathrm{Delay}=\frac{2\pi r}{\mathrm{Velocity}*Steps\;Per\;Revolution}*10^{6}$$

**Fig. 18:**
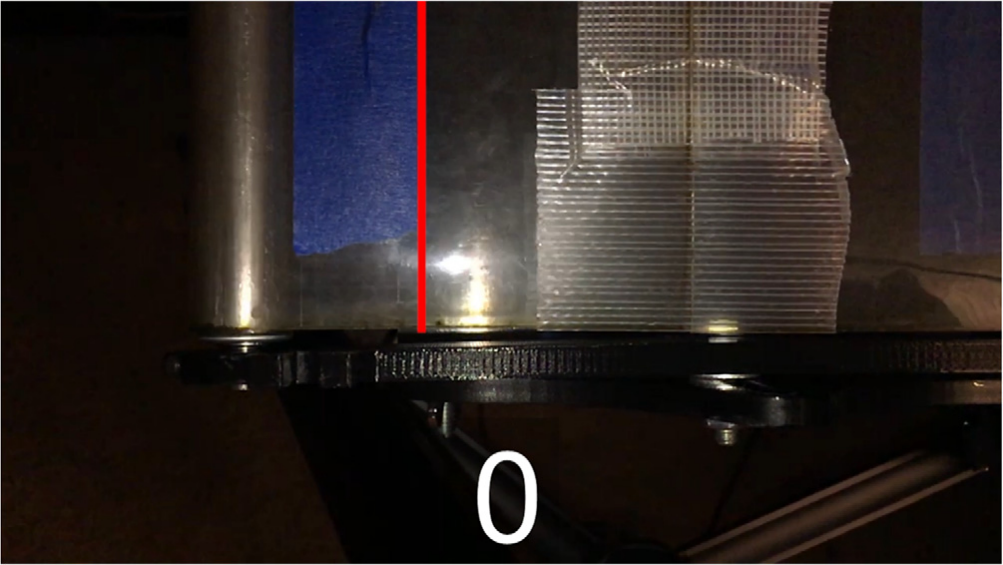
Test run start position. 0 frames have passed.

**Fig. 19:**
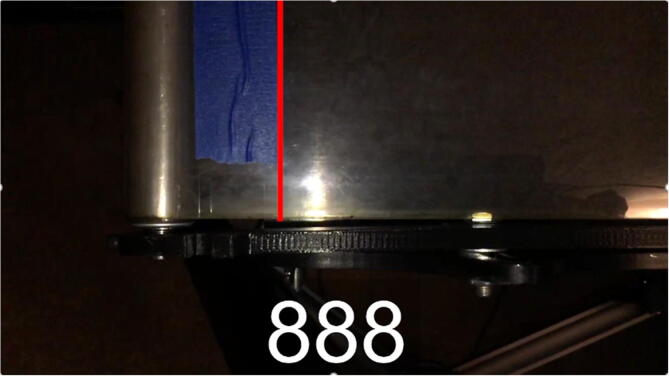
Test run end position. 888 frames have passed.

In Eq. (1), multiplying by 10^6^ converts the delay in seconds to delay in microseconds, the units used in the Arduino code. Initial tests showed that at speeds above about 12 cm/s (i.e. where the delay in microseconds became smaller, and thus required more precise timing from the Arduino) were less accurate than slower speeds. In order to verify these results and form a calibration equation, speed tests were filmed using a smartphone’s slow-motion video setting. These tests were analyzed in video editing software to count the number of frames it took the treadmill belt to travel a set distance, marked with pieces of tape (Figs. 18 and 19).

The exact time taken to travel the distance between the markers was found by dividing the number of frames by the framerate (in frames per second) of the camera.

(2) $$\mathrm{Time}=\frac{\#\;of\;Frames}{\mathrm{Frames}/Second}$$

Velocity Eqn 3 in centimeters per second was then calculated by taking the distance between the tape markers (measured in centimeters) and dividing by the time found in Eq. (2).

(3) $$\mathrm{Velocity}=\frac{\mathrm{Distance}}{\mathrm{Time}}$$

**Fig. 20:**
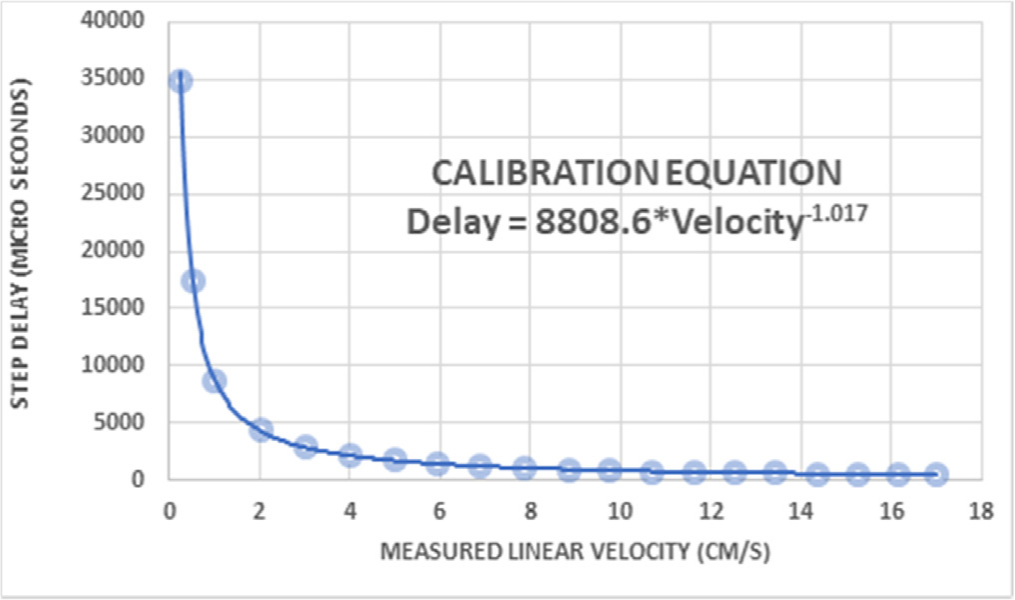
Calibration data results from speed trials.

Multiple trials were run over a wide range of velocity values. The results were then plotted to find a line of best fit (Fig. 20). The equation of this line is the calibration equation used by the Arduino to get accurate results within 0.04 cm/s. The equation already factors in the steps per revolution (800 steps) and the radius of the drive roller shaft (1.11125 cm).

This calibration allows for the following capabilities and limitations of the hardware.

### 7.1 Speed

• Operational belt speeds between 0.5 cm/s and 15 cm/s, with precision of 0.1 cm/s.
• Speed settings accurate within 0.04 cm/s.
• Belt speeds above 15 cm/s can be achieved by removing a limit in the Arduino code, but the belt speed will only be accurate within 0.25 cm/s.

### 7.2 Runtime

• Device can run a single trial for a maximum time of 10 h, with timer precision of 1 s.
• Device can theoretically be turned on and run continuously for a maximum of 40 days. (Not recommended)

### 7.3 Size capabilities

• Maximum treadmill belt area (with acrylic containment walls) roughly 20 cm × 11 cm.
• Treadmill can be placed up to 100 cm away from control device.

## 8 Recommended changes/future work

### 8.1 Recommended changes

The control enclosure could be modified to allow for more space between components and the acrylic walls. This would make construction, wiring, and maintenance of control enclosure easier, since the current design minimized the size. If a custom cut acrylic control enclosure is not possible, a pre-made box can be purchased (such as: https://www.digikey.com/product-detail/en/bud-industries/BT-2742/377-1548-ND/1640958). However, this would require a redesign of the printed internal control supports. Note that a pre-made control enclosure that is also angled is not easily available in the dimensions required for all control-components. A right-angled box can be used, and then modified with angled legs if desired.

A critical component in the design of the treadmill system was the motor selection. Originally, a Servo DC motor was selected to drive the treadmill belt. A DC motor does not require a fast microcontroller, so an Arduino MEGA was originally used to run the system. However, due to the wide range of speeds specified by the design criteria, most conventional DC motors would not have adequate torque to run at both the lowest speed (0.5 cm/s) and the highest (15 cm/s) speed, without a gearbox or transmission. The NEMA 17 stepper motor was selected to enable this wide range of speed control and eliminate the need for a gearbox. The timing precision required by both the stepper motor and the touchscreen to run in tandem demanded a microcontroller with a faster processing speed. Therefore, the Arduino DUE board was used because of its faster processing speed.

Updating the touchscreen timer requires a relatively large amount of processing power. The more pixels being changed at any moment, the more lag will be present in the system. To solve this problem, only a timer with small font on the touchscreen dynamically keeps track of the time remaining in real-time. Another possible solution is to integrate a real-time operating system (RTOS) into the code (such as FreeRTOS for Arduino) to allow both the timer and the step-function of the code to run in tandem. Another solution would be to incorporate a secondary Arduino MEGA board to run the timer, while the Arduino DUE runs the stepper motor. However, this would require large modifications to the code to split-up the functions between multiple boards.

The Arduino DUE board outputs 3.3 V from its pins, unlike the Arduino MEGA, which outputs 5 V. The components chosen were compatible with the Arduino MEGA at 5 V. For all components to be *fully* compatible with the Arduino DUE, the following components are recommended:

**NOTE: Changing components would require 3D printed supports and enclosure walls to be modified to accommodate new sizes/hole locations.**

• DM320T Stepper Driver (requires 5 V logic signal) – Replace with a stepper driver that only requires 3.3 V logic signals. Step resolution of at least 800 pulses/revolution recommended. Step resolution higher than 800 requires more precise timing, not recommended with Arduino DUE. Such as: https://www.amazon.com/STEPPERONLINE-Digital-1-4-5-6A-15-36VAC-20-50VDC/dp/B07PLTY678/ref=sr_1_5?keywords=3.3v+stepper+driver&qid=1556848702&s=gateway&sr=8-5

A lower cost, chip-type stepper motor controller could also work, such as: https://www.amazon.com/KINGPRINT-DRV8825-Stepper-Driver-Printer/dp/B075XH1TSJ/ref=sr_1_fkmrnull_5?keywords=nema+17+stepper+driver+3.3v&qid=1556848889&s=gateway&sr=8-5-fkmrnull

• DC Fan (requires 5 V) – Replace with a DC fan that requires 3–3.3 V, so it can be controlled through Arduino DUE. This would allow fan to be turned on only when trials are running. Such as: https://www.digikey.com/products/en/fans-thermal-management/dc-brushless-fans-bldc/217?k=&pkeyword=&sv=0&pv46=40589&sf=0&FV=ffe000d9%2C3800fb%2C3801a9&quantity=&ColumnSort=0&page=1&pageSize=25.

### 8.2 Future work

Future studies will use the treadmill to explore the impacts of duration (run time), intensity (variable incline), rate (variable speed) and timing (neonatal age) of exercise on tendon and musculoskeletal tissue formation in developing neonates. Prior work demonstrated that neonatal rats as young as postnatal day (P) 1 could be exposed to a moving treadmill [20]. However, at these young ages, the subject needed to be suspended over the treadmill belt. The acrylic containment walls were designed to be removable so that neonates could be suspended over the belt using the previously described soft harness system [20]. For neonates that display full weight-bearing locomotor behavior, typically beginning around P10 for rats [15], [21], [22], an external stimuli may be needed to provide motivation for treadmill running [17] and could be added to the acrylic containment walls in future studies. Additionally, based on the provided engineering drawings and controller designs, the treadmill system could be scaled-up through increased track length and motor selection for use with more mature animals.

## 9 Human and animal rights

NO human or animal studies were conducted in this work.

## Declaration of interest

The authors whose names are listed certify that they have NO affiliations with or involvement in any organization or entity with any financial interest.
